# Clinical decision support software for diabetic foot risk stratification: development and formative evaluation

**DOI:** 10.1186/s13047-015-0128-z

**Published:** 2015-12-12

**Authors:** Deborah E. Schoen, David G. Glance, Sandra C. Thompson

**Affiliations:** 1 Western Australian Centre for Rural Health, Faculty of Medicine, Dentistry & Health Sciences, The University of Western Australia, M706, 35 Stirling Highway, Crawley, 6009 WA Australia; 2 Director Centre for Software Practice, The University of Western Australia, M002, 35 Stirling Highway, Crawley, 6009 WA Australia; 3 Director Western Australian Centre for Rural Health, Faculty of Medicine, Dentistry & Health Sciences, The University of Western Australia, M702, 35 Stirling Highway, Crawley, 6009 WA Australia

**Keywords:** Diabetes, Foot ulcer, Diabetic Neuropathies, Diabetic Angiopathies, Decision Support Systems, Clinical

## Abstract

**Background:**

Identifying people at risk of developing diabetic foot complications is a vital step in prevention programs in primary healthcare settings. Diabetic foot risk stratification systems predict foot ulceration. The aim of this study was to explore the views and experiences of potential end users during development and formative evaluations of an electronic diabetic foot risk stratification tool based on evidence-based guidelines and determine the accuracy of the tool.

**Methods:**

Formative evaluation of the risk tool occurred in five stages over an eight-month period and employed a mixed methods research design consisting of semi-structured interviews, focus group and participant observation, online survey, expert review, comparison to the Australian Guidelines and clinical testing.

**Results:**

A total of 43 healthcare practitioners trialled the computerised clinical decision support system during development, with multiple software changes made as a result of feedback. Individual and focus group participants exposed critical design flaws. Live testing revealed risk stratification errors and functional limitations providing the basis for practical improvements. In the final product, all risk calculations and recommendations made by the clinical decision support system reflect current Australian Guidelines.

**Conclusions:**

Development of the computerised clinical decision support system using evidence-based guidelines can be optimised by a multidisciplinary iterative process of feedback, testing and software adaptation by experts in modern development technologies.

## Background

Diabetes is now the leading cause of lower extremity amputation in Australia [1], with approximately 85 % of lower extremity amputations in people with diabetes preceded by a diabetic foot ulcer [1–5]. The lifetime risk of foot ulceration in people with diabetes is estimated to be between 15 and 25 % [6–8]. Identifying people at risk of foot complications is a crucial step in prevention. Diabetic foot risk stratification predicts foot ulceration and has accordingly become a cornerstone of management [9].

Diabetic foot risk stratification identifies clinical features of individuals with diabetes that are predictive of the relative risk of foot ulceration in the future. A large number and type of clinical indicators including both systemic and peripheral signs and symptoms have been tested for their predictive value. Systemic features have included age, sex, weight, height, body-mass index, duration of diabetes, type of diabetes, HbA1C, fasting glucose, insulin regimes, history of myocardial infarct, hypertension, erythrocyte sedimentation rate, serum creatinine, kidney disease, eye disease, smoking and alcohol intake [10]. Peripheral features have included peripheral arterial disease, peripheral neuropathy, foot deformity, prior foot ulceration or amputation, abnormal plantar foot pressures, absent tendon reflexes, ankle-brachial index, transcutaneous oxygen tension, lower extremity bypass, intermittent claudication, tinea pedis, onychomycosis, lower leg oedema, dry or fissured skin [10]. Social factors such as level of education, occupation, socioeconomic status, religion, ethnicity and marital status have also been assessed [10–16].

Numerous diabetic foot risk classification systems are described in the literature [7, 9, 17–25]. There is strong evidence to justify risk stratification systems from large cross-sectional and prospective studies [22, 26]. The risk stratification systems have ranged from two to six risk classification groups. Monterio-Soares validated five international risk systems [7, 16, 19, 21, 22] and reported no significant difference between them and all had a high accuracy to detect people who would develop foot ulceration [27]. Most recently the international collaboration, prediction of diabetic foot ulcerations study (PODUS), of more than 16,000 people with diabetes worldwide meta-analysis reported, “the use of a 10-g monofilament or one absent pedal pulse will identify those at moderate or intermediate risk of foot ulceration, and a history of foot ulcers or lower-extremity amputation is sufficient to identify those at high risk” [28]. Notably foot deformity, ethnicity and eye disease were not included in the analysis, as they were not consistently defined in the included data sets.

As a result of expert input and review, in 2011 Australia’s National Health and Medical Research Council (NHMRC) produced *National Evidence-Based Guideline on Prevention, Identification and Management of Foot Complications in Diabetes (Guidelines)* [24]*.* This delivered a new national foot risk stratification system, consisting of three levels; low, intermediate and high risk of developing foot complications and the *Guideline* recommended ‘any trained professional may perform the risk assessment’ and urged the ‘urgent integration of decision support tools into medical software’ [24]. Research has shown that although the procedure for the assessment can be done by any trained professional, the final assessment of level of risk still proved problematic in the absence of additional decision support [29].

Clinical decision support systems in medicine have progressed from early systems that were never used in a clinical setting to systems that are now integrated into electronic health records across diverse clinical settings [30, 31]. These have been known as artificial intelligence, expert systems or clinical decision support systems (CDSS). CDSS are defined as, “any electronic system designed to aid directly in decision making, in which characteristics of individual patients are used to generate patient-specific assessments or recommendations that are then presented to clinicians for consideration” [32]. They prompt clinicians through a protocol of pertinent, evidence-based clinical actions and decisions to enhance health-related decisions and actions. CDSS support healthcare professionals to work at a higher level than their standard scope of practice. This is required when there is a workforce shortage of relevant expertise [33], as occurs in Western Australia where a shortage of podiatrists in rural and remote areas is recognised [34, 35]. Successive systematic reviews have shown the beneficial effect of CDSS on clinical decision making to improve practitioner performance in diagnostic systems, reminder systems, chronic disease management processes of care, drug dosing or prescribing systems, improve rates of screening, and improve adherence to recommended care standards [32, 36–41]. CDSS are increasingly considered to be one of the most effective instruments to improve guideline implementation [38, 42–44]. Trials of CDSS including diabetic foot processes of care have all shown improvements in practitioner performance, rates of screening and adherence to guidelines [45–51]. Most recently, Moja’s systematic review of new generation CDSS in electronic health records reported no reduction in patient mortality but concluded that they might moderately decrease morbidity [31]. Obstacles to wider use of CDSS include poor usability or integration into practitioner workflow, failure to integrate with primary care information technology, the attitudes of end-users, practitioner non-acceptance of computer recommendations, lack of clinician input into the development and their failure to fulfil a perceived clinical need [38, 52, 53].

Diabetic foot risk stratification lends itself to CDSS because it has a strong evidence base, is unambiguous, has explicit input and output criteria for each risk stratification level, each foot risk choice is binary, every possible permutation of foot risks can be decided by an algorithm and produces a correct result. The Scottish Care Information – Diabetes Collaboration system’s diabetic foot electronic decision support tool is a CDSS and has been validated and shown to be predictive of ulceration [22].

In this study we sought to act on the NHMRC *Guideline* to integrate CDSS into Australian electronic health records to ensure it fitted within healthcare professionals’ usual workflow [24]. The underpinning question related to whether the use of a CDSS could assist non-podiatrists in risk stratification. The importance of a usable and accurate foot risk assessment CDSS is underpinned by the shortage of podiatrists in rural and remote areas of Western Australia [34, 35].

This paper reports on the process of usability testing that was conducted to develop and evaluate an electronic diabetic foot risk stratification tool for its use in the treatment and management of diabetes in a largely Aboriginal population in Western Australia. The aims of the usability testing were to not only produce a system that was clinically correct, but one that would fit within and enhance the workflow of clinical practice of the health professionals concerned.

## Method

### Risk tool design

A podiatrist at the Western Australian Centre for Rural Health (DES) and software developers at the Centre for Software Practice at The University of Western Australia (led by DGG) collaborated to develop the risk tool. It was designed to be used by both podiatrists and non-podiatrists for assessing foot risk in people with diabetes in the primary healthcare setting. A usability design process was used incorporating an early focus on users, integrated design, early and continual user testing and iterative design [54]. The term ‘usability’ means getting ‘real’ people to test the system via interviews, observations, surveys. This approach intertwined design and evaluation as users’ feedback was iteratively used to make design changes and improve the system. The risk tool is nested in the cloud-based patient information record system MMEx, which was developed by The Centre for Software Practice in 2007 [55]. MMEx is an e-Health platform providing electronic health records, secure messaging, collaborative care and evidence-based forms with clinical decision support available on mobile platforms. MMEx has been adopted by a broad range of primary, secondary and tertiary public healthcare services, Aboriginal Community Controlled Health Organisations and private practices in several jurisdictions of Australia.

**Fig. 1:**
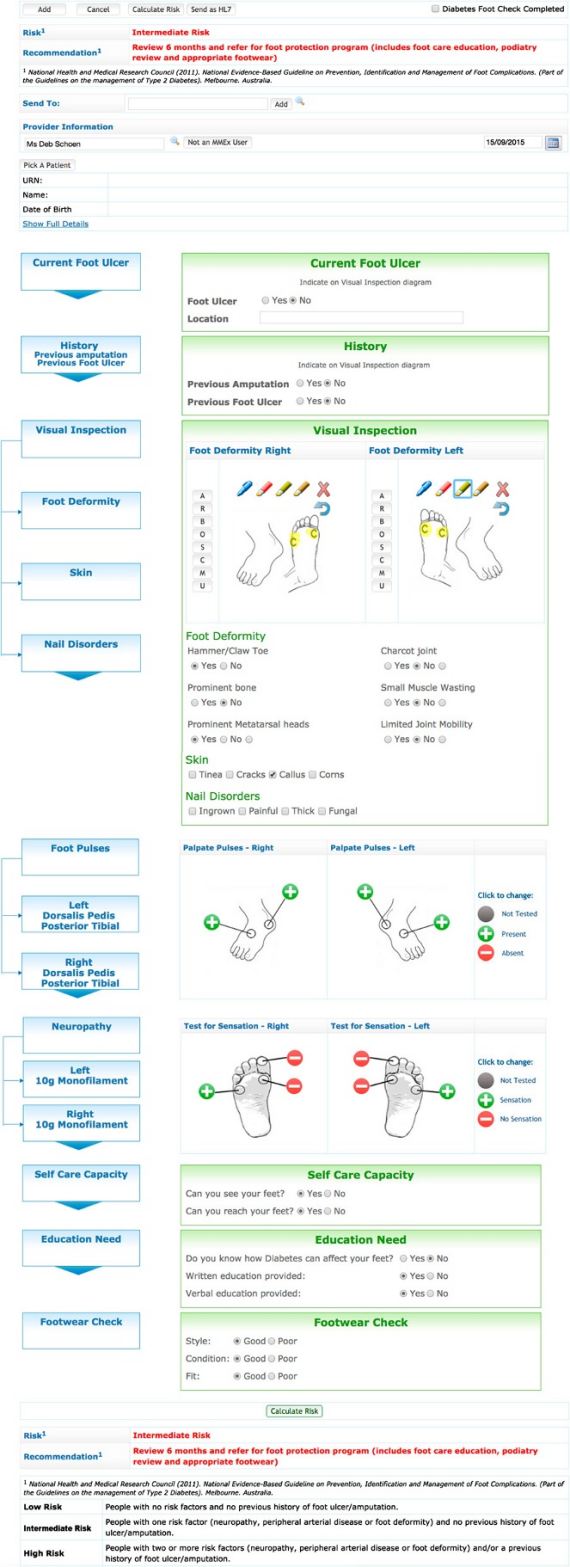
The risk tool

The risk tool is based on the NHMRC *Guidelines* clinical inputs of (1) previous amputation, (2) previous foot ulcer, (3) foot deformity, (4) pulses, and (5) peripheral neuropathy [24]. The CDSS risk output appears as the words low, intermediate or high risk and is accompanied by the NHMRC recommendation for each given level of risk [24]. For example, intermediate risk suggests “Review 6 months and refer for foot protection program (includes foot care education, podiatry review and appropriate footwear” (Fig. 1).

The NHMRC *Guidelines* were transferred into an online format with CDSS in two steps [24]. Initially, a series of ‘yes/no’ flowcharts of the five clinical inputs was created to make the decisions to decide the risk output. Next, a score to determine the level of risk was assigned with ‘No’ responses scored as zero and ‘yes’ responses scored as one point. If the five risk inputs are all no, then the total score was zero and the risk output was determined as ‘low risk’; if the five risk input score equalled one then the risk output was ‘intermediate risk’; if the risk input score was greater than one, then the risk output was ‘high risk’. Additionally, the presence of a current foot ulcer as a risk input was scored as greater than one so that the output was always high risk. This logic was the basis of the computer code in the development of the tool. The last three elements of the risk tool were practical risk factors that need to be considered in foot documentation although they had not been determined as essential elements of determining the patients’ level of risk. These were the ability to see and reach feet, assessment of footwear and recording of diabetic foot education provided. Their inclusion was to guide clinical practice and prompt health professionals to undertake recommended tasks such as providing written diabetes education or advice on practical risks that need to be considered and addressed.

### Phased formative evaluation and iterative development of the risk tool

#### Study design

Formative evaluation of the risk tool occurred in five stages over an 8-month period and employed a mixed methods research design consisting of semi-structured interviews, focus group and participant observation, online survey, expert review, comparison to NHMRC *Guidelines* and clinical testing [56]. Concurrent qualitative and quantitative data collection procedures were used. Seven healthcare professionals participated in one-to-one semi-structured interviews and observations in Phase One. The second phase was quantitative practical live testing in a routine clinical setting using real patients by the first author (DES) on a tablet in a private rural podiatry practice in Western Australia. The time taken to complete the risk tool within a clinical appointment, language, design and the accuracy of the calculated risk and recommendation were evaluated. Phase Three involved a series of one-to-one semi-structured interviews, observation and one focus group with local experts and potential end users. Twenty local health practitioners (one vascular surgeon, seven podiatrists, four allied health, three general practitioners, three nurses, one nurse practitioner and one Aboriginal Health Worker) participated in one-to-one interviews. They were given web-based access, asked to work with the risk tool, observed during their use and then interviewed. Supplementing this was a focus group discussion with eight diabetes educators. The fourth phase was a quantitative national review of the risk tool by three podiatrists from different states of Australia, who completed an online survey. It contained four scenarios to ascertain if the calculated risk and recommendation were accurate. The participants responded to questions regarding the language, design, and a request for suggestions for improvement of the risk tool. The fifth phase of the evaluation was web-based review by four experts outside of Australia with a publication record in diabetic foot risk stratification systems. They appraised the system for suitability of key criteria and suggestions for improvement.

Ethics approvals for this study were granted by the University of Western Australia, the Western Australian Aboriginal Health Ethics Committee and the Western Australian Country Health Service.

#### Study population and setting

A total of 43 healthcare professionals participated, 26 novice and 17 experts. Seven in Phase One, one in Phase Two, 28 in Phase Three, three in Phase Four and four in Phase Four. The study was coordinated from Western Australia; Phases One and Two were in rural Western Australia, Phase Three in both rural and urban settings, Phase Four involving participants in three Australian states and Phase Five utilising international reviewers.

#### Sampling

Purposive sampling of local healthcare professionals as potential end users of the risk tool and local, national and international experts in diabetic risk stratification systems participated.

#### Data collection

Written notes were taken during semi-structured interview and observation of participants’ actions, hesitations or when they needed prompting to complete the risk tool and answers to questions. Live testing risk errors, as judged by the first author based on the NHMRC *Guidelines*, were recorded in MMEx [55].

#### Data analysis

Participants’ comments and observed actions or inaction were thematically categorised into language, design, workflow or risk errors. These were then translated into concrete actions for modification by the MMEx software development team. All suggestions were cross-checked against NHMRC *Guideline* [24]*.*

## Results

### Phase 1: Results of one-to-one interviews and observations

Participants were all supportive of the risk tool concept. Positive comments were that the language was suitable, it was simple to use, took minimal time to find within the electronic patient information system, and it was easy to complete the risk tool. Participants suggested the recommendation for the given level of risk should be displayed at the completion of the risk assessment information being entered.

### Phase 2: Live testing

Two hundred and ninety-nine individual risk assessments were completed in a real clinical setting. The risk tool was able to be completed in less than 10 minutes making it suitable for a clinical appointment. The CDSS calculation was fast, taking less than 1 second to calculate the risk and recommendation. Live testing revealed risk errors due to the foot deformity score not initially being calculated and this required further refinement by the MMEx development team. The workflow of the risk tool matched a standard diabetic foot assessment by following the general medical sequencing of a clinical examination process of history, inspection, palpation then specialised procedures with the monofilament. This process also encouraged good infection control procedures to be followed as the history and visual inspection could be completed without gloves on so that interaction with the computer or tablet occurred before donning gloves for the physical assessment. After completing the physical tasks of the risk assessment, hands would be cleaned appropriately and the rest of the tool completed. The self-care and education questions were found to be difficult to deliver without leading the patient in any response/direction and, the need to refine this part of the tool led to initiating the focus group with diabetes educators.

### Phase 3: Results of one-to-one interviews and observations

Five language concerns were raised. Three concerns were easily solved by correcting spelling, removing subjective qualifiers (e.g. very/superficial) and incorrect definitions. The other two issues were terminology. Participants felt “Send as HL7” was meaningless to the user, and queried its necessity. Secondly, they suggested the “high risk” recommendation should be “Refer to a High Risk Foot Podiatrist.” Participants also suggested a free text section was needed. A major workflow issue was exposed by observation of participants. They were unable to complete the risk tool as the “Calculate Risk” button was at the top of the risk tool only and resulted in participants thinking the CDSS had not worked. A critical design flaw of the CDSS was revealed. A false negative error occurred if the entire risk tool was not completed. A false negative is an error in which a test result improperly indicates no presence of a condition (the result is *negative*) when in reality it is present. This occurred during testing if one section, for example, pulses, was not completed and results in an incorrect risk stratification (Table 1).

**Table 1:** Participant feedback from Phase three

Evaluation	Participants	Methodology used	Feedback	Changes made
Phase three	1 VS 7 Podiatrists 4 AH 2 Nurses 1 NP 1 AHW 8 DE 8 Experts and 20 Novices	Web-based review One-to-one interviews Observation Focus group	1. Risk error false negative	1. Forced completion of all sections
			2. Language a) HL7 b) Spelling c) Subjective terms d) Incorrect definitions e) Terminology	2. Language a) Unchanged, important b) Corrected c) Removed d) Removed e) Unchanged
			3. Workflow a) Risk display at bottom of form b) Send at bottom of form c) Boxes around each section	3. Improved Workflow a) Changed b) Unchanged/can be incorporated c) Unchanged
			4. Questions a) Self-care questions too vague b) Education questions objective c) Education questions not specific foot knowledge	4. Questions a) Simplified; two direct questions b) Simplified; one direct question c) One specific foot question
			5. Suggestions a) Opportunistic education within design b) Free text section	5. Suggestions a) Unchanged/can be incorporated b) Unchanged/can be incorporated
			6. Dislikes a) Too many pens in foot deformity b) Letters in foot deformity c) Don’t call it deformity	a) Unchanged b) Unchanged c) Unchanged

*VS* vascular surgeon, *AH* allied health, *NP* nurse practitioner, *AHW* Aboriginal Health Worker, *DE* diabetes educator

### Phase 3: Results of focus group with diabetes educators

Diabetes educators highlighted that the three self-care and three education questions were ambiguous and subjective. They suggested both sets of questions should be changed from the third to the second person point of view and directly ask patients the questions. They suggested that self-care be reduced to two clear questions, directly asking the patient if they could see and reach their feet. The education question was recommended to be simplified to one question only, asking the patient what they understood. This would enable education to be directed to any identified deficits in further foot care education. Additionally, they suggested the clinician should document if verbal or written, or both forms of education were provided. Their final suggestion to improve the tool was to use the features within the form design for opportunistic education, for example, pictures of foot deformities.

### Phase 4: Quantitative national review

National experts confirmed the accuracy of the risk stratification output. However, the recommendations were incomplete and not the exact wording of the NHMRC *Guideline* [24]. The language of the risk and recommendation for a current ulcer was problematic. They suggested the risk and recommendation be referenced and a web link to the NHMRC *Guidelines* be included [24]. National experts suggested the workflow could be altered, to have the assessment of pulses and neuropathy sections first, given their greater importance. They also suggested having a free text section. Positive comments are shown in Table 2.

**Table 2:** Positive participant feedback

“…. such a simple, clear, user-friendly tool that is evidence based….”
“Also liked the pulses and neuro + and – buttons, easy to use, but also you’ve made it slow enough so as to limit errors with too many click - well done!”
“I think it’s wonderful and love the concept and design. ….has the potential to be used very widely…..”
“Lovely, enjoyable and useful tool”

### Phase 5: International review

International reviewers suggested the current foot risk should be more prominent and include an automatic recall for when the next foot assessment is due. Two experts considered the foot deformity section of the form to be too large and to contain elements (e.g. small muscle wasting) that were unlikely to inform risk assessment. Both inclusions of vibration perception threshold to improve sensitivity and specificity of the tool and, adding eschar to foot deformity and rockerbottom to Charcot foot were suggested. Additionally, they suggested the tool be printable to only one page. Finally, they also suggested using features within the risk tool design for opportunistic education, defining the variables or “Help” section. Positive comments received are included in Table 2.

## Discussion

Diabetic foot risk stratification with CDSS can be integrated into an electronic health record with minimal impact on a podiatrists’ usual workflow. A usability design process with an early focus on end users, integrated design and early and continued user testing was important in recognising major usability issues. Participants’ qualitative responses confirmed the language is suitable, generated extensive feedback for improvement and revealed essential design flaws. Live testing and participants’ quantitative results confirmed the accuracy of the tool. The resultant risk tool is fast, accurate, compatible with the workflow of a diabetic foot assessment and free from false negative errors of risk stratification based on the 2011 NHMRC *Guidelines* [24].

The mixed methods approach used in this study has been advocated in the development and evaluation of CDSS [57–63]. This approach was appropriate given the obstacles recognized in the literature that limit the use of CDSS. User-centered design processes can help improve usability and result in more likable computer applications [54, 64]. The naturalistic design of the live testing with real patients for field tests is suggested by Kaplan [65] and was the approach used to validate the Scottish Care Information – Diabetes Collaboration system’s diabetic foot electronic decision support tool [22]. Measurement of accuracy by peer review and comparison to a standard guideline avoids circularity, and the completeness of results can be supported by triangulation of data from complementary methods comparing data from semi-structured interviews, participant observation, online survey responses, NHMRC *Guidelines* and clinical testing [57, 60, 62].

Other studies have shown that major usability flaws in CDSS can be recognised by all methods regardless of the expertise of the evaluator as they rely on observation and are easy to perform [53, 66, 67]. Kilsdonk demonstrated a user-centred CDSS design can overcome usability problems when replacing paper-based clinical guidelines into an online format with CDSS as we have done in this study [64]. The Scottish Care Information – Diabetes Collaboration system’s diabetic foot electronic decision support tool, used similar evidence-based guidelines to implement a diabetic foot risk stratification tool in a central web-based database opposed to integrated into an electronic health record as we have done [22]. Our risk tool aligns with Curran’s findings of the clinical reasoning and diagnostic procedures of novice podiatrists of visual cues, touch cues, questions for the patient, and then a diagnostic statement by the podiatrist [68].

The implications of non-podiatrists using the risk tool have been considered, as CDSS allow healthcare practitioners to work at a higher level of expertise. Studies report that non-podiatrists overreport the presence of foot deformities [29, 69] and are unable to palpate reliably pedal pulses [22, 29, 69]. This would result in a higher risk stratification and is safe for patients with diabetes as the NHMRC recommendation for the higher risk is for referral to a podiatrist for second stage assessment [24]. The recent PODUS study has shown the consistent reliability of the 10-g monofilament regardless of the expertise of the tester, the number of sites and the anatomical sites tested based on five different studies and 11,522 people from three different countries [28]. Finally, the combination of clinical tests for integrated foot risk score as we have used in this study is more sensitive than individual clinical tests for predicting foot ulceration [24, 70, 71].

The strengths of this study were collaboration with an experienced development team, early input from multiple users; the mixed methods approach, and development within a well-established patient information record system [55]. Limitations of this study are the small sample size, no assessment of intraobserver reliability, and that formal usability techniques such as talk aloud protocols or walkthroughs were not used. Furthermore, this study reports the formative evaluation of the risk tool only. Further summative evaluation on the complete system is warranted [62] and prospective observational studies to determine if the risk tool accurately predicts foot ulceration in a primary healthcare setting.

## Conclusion

This study illustrates that the views and experiences of potential end users can be used effectively to develop and evaluate a diabetic foot risk tool with CDSS based on evidence-based guidelines integrated into an electronic health record. The risk tool integrates simple assessment readily available in a clinical setting, and should have minimal impact on experienced healthcare professionals’ usual workflow and reflects current Australian guidelines [24]. It also structures foot examination for those who are less proficient in diabetic foot assessment and will ensure that identified predictors of risk for foot ulcers are referred in a more timely way for definitive assessment and management.
